# Soil water uptake from different depths of three tree species indicated by hydrogen and oxygen stable isotopes in the permafrost region of Northeast China

**DOI:** 10.3389/fpls.2024.1444811

**Published:** 2024-10-04

**Authors:** Biao Li, Xiaodong Wu, Xingfeng Dong, Haoran Man, Chao Liu, Siyuan Zou, Jianxiang He, Shuying Zang

**Affiliations:** ^1^ Heilongjiang Province Key Laboratory of Geographical Environment Monitoring and Spatial Information Service in Cold Regions, Harbin Normal University, Harbin, China; ^2^ Heilongjiang Province Collaborative Innovation Center of Cold Region Ecological Safety, Harbin, China; ^3^ Cryosphere Research Station on the Qinghai-Tibet Plateau, State Key Laboratory of Cryospheric Science, Northwest Institute of Eco-Environment and Resources, Chinese Academy of Sciences, Lanzhou, China

**Keywords:** climate change, vegetation growth, permafrost degradation, xylem water, mixing model

## Abstract

Global warming has caused the gradual degradation of permafrost, which may affect the vegetation water uptake from different depths. However, the water utilization strategies of different vegetation species during the thawing stages of permafrost regions need further study. To elucidate these differences, we selected the permafrost region in Northeast China as study area. We analyzed the water uptake from different depths of *Larix gmelinii*, a deciduous coniferous tree, *Pinus sylvestris* var. *mongolica*, an evergreen tree, and *Betula platyphylla*, a deciduous broadleaf tree, using stable isotopes of xylem water, soil water, and precipitation from June to October 2019. The results showed that *L. gmelinii* primarily used shallow soil water (0-40 cm) with the highest proportion at 64.1%, *B. platyphylla* generally used middle soil water (40-110 cm) with the highest proportion at 55.7%, and *P. sylvestris* mainly used middle (40-110cm) and deep soil water (110-150 cm) with the highest proportion at 40.4% and 56.9%. The water sources from different depths exhibited more frequent changes in *P. sylvestris*, indicating a higher water uptake capacity from different soil depths. *L. gmelinii* mainly uptakes water from shallow soils, suggesting that the water uptake of this species is sensitive to permafrost degradation. This study revealed the water uptake strategies from different depths of three tree species in a permafrost region, and the results suggested that water uptake capacity of different tree species should be considered in the prediction of vegetation changes in permafrost regions under a warming climate.

## 1 Introduction

Forests pivotal in regulating global energy flows, hydrology, and carbon cycle have their distribution, biomass, and abundance are predominantly governed by temperature and water resources (Dodd et al., 1998; Drake and Franks, 2003; Tao et al., 2016). Water use strategy is an important mechanism used by plants to adapt to the environment and is a key controlling factor for the species distribution (Wang et al., 2017). The capacity of plants to uptake water from rainfall, soil water, runoff water, and groundwater is largely determined by the distribution of their roots (Sun et al., 2011). Generally, Herbaceous plants predominantly utilize water from shallow soil layers (Asbjornsen et al., 2008; Priyadarshini et al., 2016), while shrubs and trees are able to utilize deep soil water sources (Mccole and Stern, 2007; Wu et al., 2016). The differences in water use strategies may be a mechanism for the coexistence of various vegetation species (Wu et al., 2016).

Global warming has led to the gradual degradation of permafrost, e.g., increase soil temperature and the active layer thickness (Cheng and Wu, 2007; Biskaborn et al., 2019; Chen et al., 2023; Zhang et al., 2023). The interactions between permafrost and vegetation are very complicated. Vegetation can affect the underlying permafrost hydrothermal conditions via the mulching effect, soil heat convection, evapotranspiration, and water retention capacities on the one hand (Tchebakova et al., 2009), permafrost changes can also impact the vegetation growth in the permafrost regions via the regulation of soil water, temperature, and nutrients supply on the other (Dong et al., 2021). Among these mechanisms, plant roots are crucial for the uptake of water and nutrients. In permafrost regions, the majority of the vegetation roots are distributed in the active layer (Blume-Werry et al., 2019). In areas where permafrost has been degraded, deep roots can also invade newly thawed permafrost located underneath, and these changes will inevitably affect the water uptake capacity of plants (Blume-Werry et al., 2019).

In cold regions, plant water uptake from different depths exhibits a seasonal variation due to the freeze-thaw cycle of soils. In early spring, surface soils begin to thaw, making liquid water available for plant growth. During the summer, soil water in the thawed active layer can be utilized for plant growth (Sugimoto et al., 2002). Soil water derived from rainfall infiltration typically constitutes the principal resource for plant growth (Asbjornsen et al., 2008); however, water released from thawing permafrost becomes a critical source when losses due to soil evapotranspiration surpass precipitation (Churakova-Sidorova et al., 2020). During the freezing period, soil freezes rapidly and the liquid water content is low (Dong et al., 2021). At this stage, the majority of the vegetation remains dormant and the water utilization rate is low (Tiemuerbieke et al., 2018). The period of vegetation growth predominantly occurs during the thawing phase of perennial permafrost. Thus, it is imperative to examine vegetation water use strategies within this critical phase.

Multiple methods are available to assess the water use status of vegetation, such as root system excavation (Swaffer et al., 2014), radioactive tracer tritium (Schwendenmann et al., 2015). However, these methods tend to be time-consuming, costly, and even radioactive. Scholars have recently proposed the use of oxygen isotopes to identify water resources (Ding et al., 2021; Ma et al., 2021; Sun et al., 2024),as isotopic fractionation does not occur before water absorbed by plant roots is transported to the leaves through the xylem. The quantification of plant water sources can be achieved by analyzing hydrogen and oxygen isotope ratios in xylem water and potential sources, employing the MixSIAR model (Huo et al., 2018; Ma et al., 2021).

From the 1950s to the 2010s, the degradation of permafrost in northeastern China has become more pronounced, leading to a decrease in the total area from 4.8 × 10^5^ km² to 3.1 × 10^5^ km² (Zhang et al., 2021). The Greater Khingan Mountains in Northeast China is the largest area of virgin natural forests, which is sensitive to climate change due to the fragility of the permafrost environment. A diverse array of vegetation thrives, with the majority of root systems concentrated within the active layer (Blume-Werry et al., 2019). The continuing degradation of permafrost may affect plant water uptake as it can induce a deeper active layer and longer thaw period (Oliva and Fritz, 2018; Li et al., 2022). These changes may further influence plant survival and vegetation succession, given that water utilization is a critical determinant of plant growth (Wang et al., 2017). However, most of the current research on vegetation water sourcing focuses on arid, semi-arid, and watershed regions, leaving a significant knowledge gap regarding the water absorption strategies among different vegetation types during the thawing stages of permafrost regions. These knowledge gaps thus limit our insights into the future dynamics of plant species in permafrost regions under a warming climate.

In this study, we focused on the permafrost region of the Greater Khingan Mountains in Northeast China, investigating the water sources and utilization strategies of three tree species, namely *Larix gmelinii* (deciduous coniferous tree), *Pinus sylvestris* var. *mongolica* (evergreen tree) and *Betula platyphylla* (deciduous broadleaf tree). We analyzed the stable hydrogen and oxygen isotopic compositions of xylem water, soil water and precipitation during June-October 2019. The results can provide insights into the effects of permafrost degradation on vegetation growth and provide some scientific reference for the prediction of vegetation changes with future climate in the permafrost regions.

## 2 Materials and methods

### 2.1 Study area

**Figure 1:**
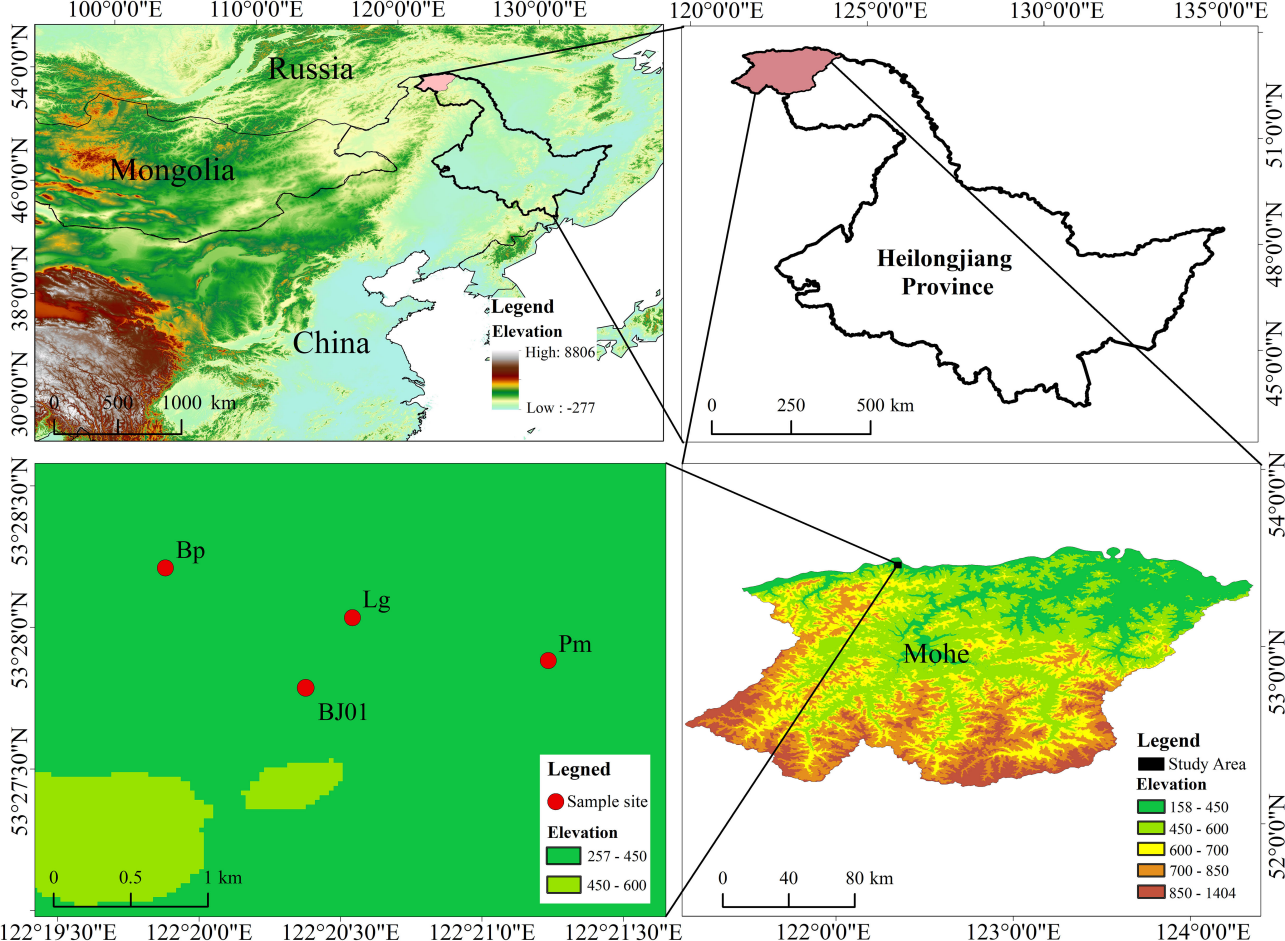
Study area in the Mohe, Heilongjiang Province (Lg is the sampling site for *Larix gmelinii*, Pm is the sampling site for *Pinus sylvestris* var. *mongolica*, Bp is the sampling site for *Betula platyphylla*, BJ01 is the sampling site for soil water content, air temperature and humidity, water vapor pressure, and ground temperature).

The study area is located in close proximity within Beiji Village, Mohe City, Heilongjiang Province, in the northern Greater Khingan Mountains (52°10′-53°33′N, 121°07′-124°20′E) (**Figure 1**) and belongs to mountainous permafrost regions. The region is characterized by an average elevation ranging from 300 to 500 meters and experiences a cold-temperate continental monsoon climate. The tree species mainly include *Larix gmelinii*, *Pinus sylvestris* var. *mongolica*, *Betula platyphylla*, etc. Shrub species include *Vaccinium uliginosum, Rhododendron dauricum, Ledum palustre*, etc., and the dominant herbs are *Pyrola incarnata*, *Eriophorum vaginatum*, etc.

Sample trees were selected according to mean average diameter at breast height (DBH, 1.3 m) and height (H). The average DBH of *L. gmelinii, P. sylvestris* and *B. platyphylla* were 18.4 cm, 25.1 cm and 16.8 cm, with heights of 16.5 m, 20.3 m and 15.1 m. Three replicates were taken from each tree for the measurement of stable hydrogen and oxygen isotopes.

### 2.2 Sample collection

Monthly sampling might reflect changes in vegetation water use strategies (Asbjornsen et al., 2008). However, since the active layer of permafrost remained predominantly thawed from June to October, with significant soil water variation across periods, we opted for semi-monthly sampling to more effectively capture changes in plant water uptake strategies. In 2019, Vegetation xylem, soil, and precipitation samples of *L. gmelinii, P. sylvestris* and *B. platyphylla* were collected on June 8, June 20, July 7, July 26, August 7, August 26, September 10, September 26, and October 6 (total of nine sampling dates). To mitigate the effects of light, temperature and other environmental variables on isotope fractions, sampling was conducted from 08:00 to 10:00 local time. Samples were preserved at low temperatures in an insulated box with ice and promptly transported to the laboratory for analysis.

The branches of *L. gmelinii, P. sylvestris* and *B. platyphylla* (from the east, west, north, and south) were harvested using high branch shears to a length of approximately 5-8 cm. The tree bark and phloem of the branches were quickly peeled off and placed into a 10 mL glass vial. The cap was securely tightened and the vial was sealed using a sealing film. The total number of vegetation xylem samples collected was 81. Soil samples were extracted using a gasoline-powered auger at 10 cm intervals from the surface down to 150 cm depth. These samples were utilized to analyze the hydrogen and oxygen isotopic compositions in soil water. Overall, 405 soil samples were collected. In each sampling area, precipitation collectors were installed, featuring a 200 mm diameter funnel with a Ping pong ball to inhibit evaporation. Collected precipitation was stored in 500 ml bottles (Wang et al., 2010). Then, Samples were decanted into 50 ml airtight polyethylene vials, subsequently filtered through a 0.22 μm membrane, transferred to 2 ml screw-top vials, sealed with Parafilm, and stored at 4°C in a frozen state for hydrogen and oxygen isotope analysis. In total, 24 precipitation samples were collected.

### 2.3 Isotope measurement

Water was extracted from vegetation xylem and soil samples using an automated vacuum condensation extraction system (LI-2100). Subsequently, the extracted water and precipitation samples were subjected to hydrogen and oxygen isotope measurements using an isotope mass spectrometer MAT253. The δ^18^O and δD are expressed as permillage relative to Vienna Standard Mean Ocean Water (V-SMOW):


(1) $$\text{δ}=[({Ｒ}_{\text{Sample}}/{Ｒ}_{\text{Standard}})-1]\times 1\;000‰$$

where R_Sample_ is the ratio of the heavy isotopes to light isotopes in water samples; and δ^18^O and δD are ^18^O/^16^O and ^2^H/^1^H, respectively.

### 2.4 Soil and meteorological data

The data used in this study include soil water content (θ), air temperature and humidity, water vapor pressure (WVP), and ground temperature (Ts). The unfrozen water content, denoted as θ, was determined using a Hydra soil moisture sensor, achieving an accuracy of ±3%. Ts was measured using a 105T thermocouple probe, which offers a precision of 0.1°C. The probe was installed at 5, 10, 20, 40, 60, 80, 100, 120, 130, 150 cm in vertical profile. The air temperature and humidity were recorded using the HC2-S3 air temperature and humidity sensor. These sensors were interfaced with a CR1000 data logger, capturing data at 30-minute intervals.

### 2.5 Direct comparisons method and MixSIAR model

We conducted a qualitative analysis of the water sources for the three vegetation species using the direct comparison method. Oxygen isotopes from the potential water sources were compared with those from the vegetation xylem, with the overlap or proximity between the two considered indicative of the primary water source for the vegetation-the closer the match, the greater the proportion of water absorbed and utilized by the vegetation (Laur and Hacke, 2014; Yang et al., 2015). However, this method only provides a preliminary identification of the primary absorption layer and does not quantify the contributions of each potential water source to the vegetation’s water uptake.

The two-end-member mixing model and MixSIAR model can quantify the proportional contribution of each potential water source to vegetation water uptake (Rossatto et al., 2014; Song et al., 2016; Wang et al., 2017). The former generally requires only two potential water sources, while the MixSIAR model can accommodate three or more. Additionally, MixSIAR enhances the performance of fixed and random effects, processes errors and residuals, and integrates sources of uncertainty (Stock et al., 2018). In our study, due to the involvement of multiple potential water sources, we employed the MixSIAR model to quantify the contributions of each to vegetation water uptake. We used the average δD and δ^18^O values of xylem water from the three vegetation species across different periods, along with the average δD and δ^18^O values of each potential water source, and fractionation data (defaulted to 0, assuming no fractionation) as model inputs. The runtime for Markov chain Monte Carlo (MCMC) was set to ‘normal’ (chain length = 100,000; burn = 50,000; thin = 50; chains = 3), “Process only” was used for the error selection. Before output, model convergence was confirmed using Gelman-Rubin and Geweke diagnostics. To reduce errors in the analysis process, the water sources were divided into three potential depths (Huo et al., 2018):

1. in the shallow soil water layer (0-40 cm), the δ^18^O values are unstable and most enriched.
2. in the middle soil water layer (40-110 cm), the δ^18^O values exhibit limited relative variation.
3. in the deep soil water layer (110-150 cm), the δ^18^O values are relatively uniform and relatively depleted.

### 2.6 Data analyses

We conducted ordinary least squares (OLS) regression between δD and δ^18^O values from precipitation, xylem water of different tree species, and soil water to derive the local meteoric water line, xylem water lines, and the soil water line in the study area. A one-way ANOVA, followed by Tukey’s *post-hoc* test (p< 0.05), was employed to assess differences in δD and δ^18^O values among the xylem water of different tree species. Statistical analyses were performed using SPSS 19 (IBM), and all graphs were generated with Origin 2018 (OriginLab).

## 3 Results

### 3.1 Climate conditions

**Figure 2:**
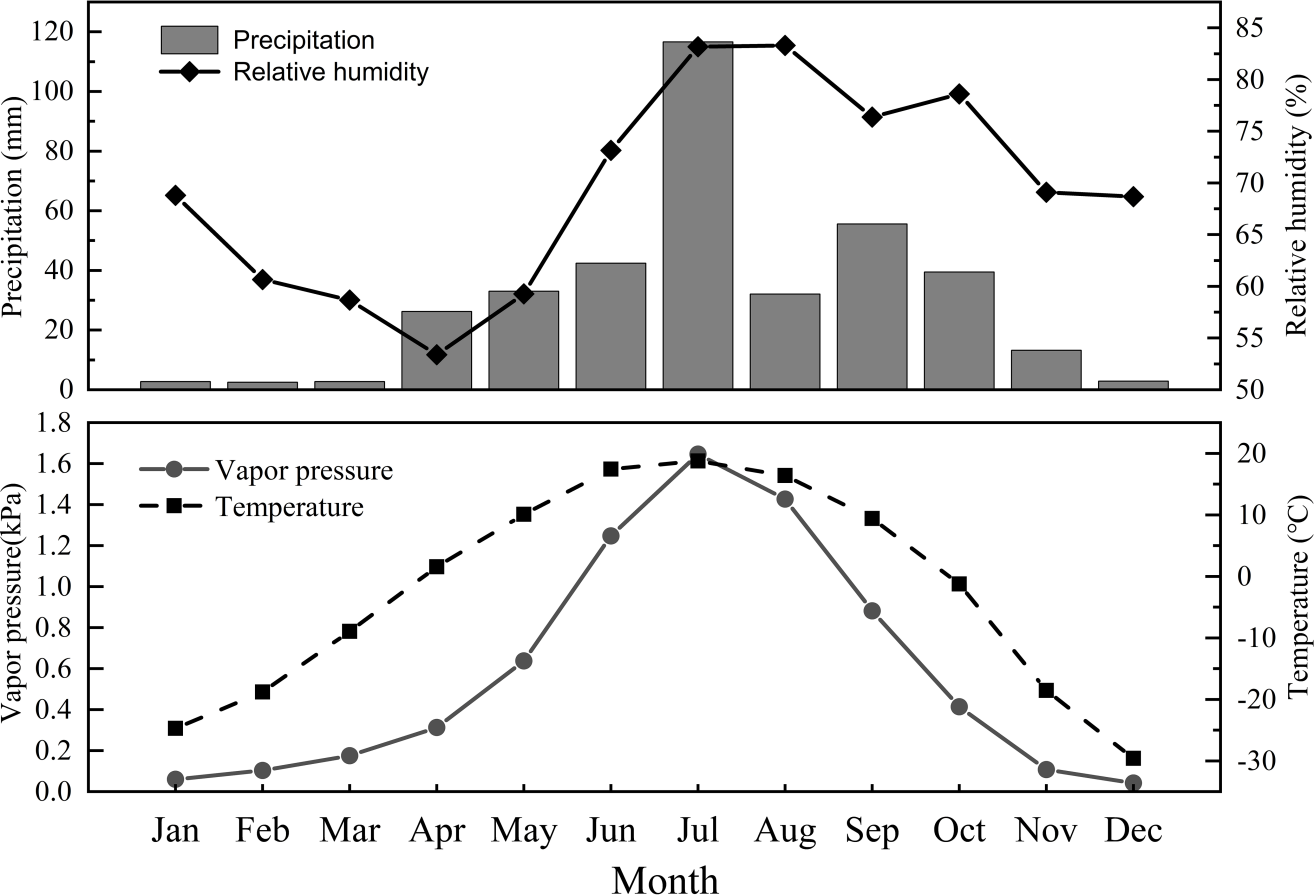
Variation of precipitation (mm), air temperature (°C), relative humidity (%) and water vapor pressure (kPa) in the study area during 2019.

The annual temperature in the study area in 2019 ranged from -39.59 to 32.72°C, with a mean annual air temperature of -2.35°C. The total precipitation was 369.4 mm, with monthly averages of 42.4, 116.6, 32.1, 55.6, and 39.5 mm from June to October, respectively. The overall trends in variation of precipitation, air temperature and water vapor pressure over time were similar, initially increasing and subsequently decreasing. The relative humidity showed an overall trend of initially decreasing, then increasing, and subsequently decreasing. All four indicators reached their maximum values in July (**Figure 2**).

### 3.2 Soil temperature and soil water content

**Figure 3:**
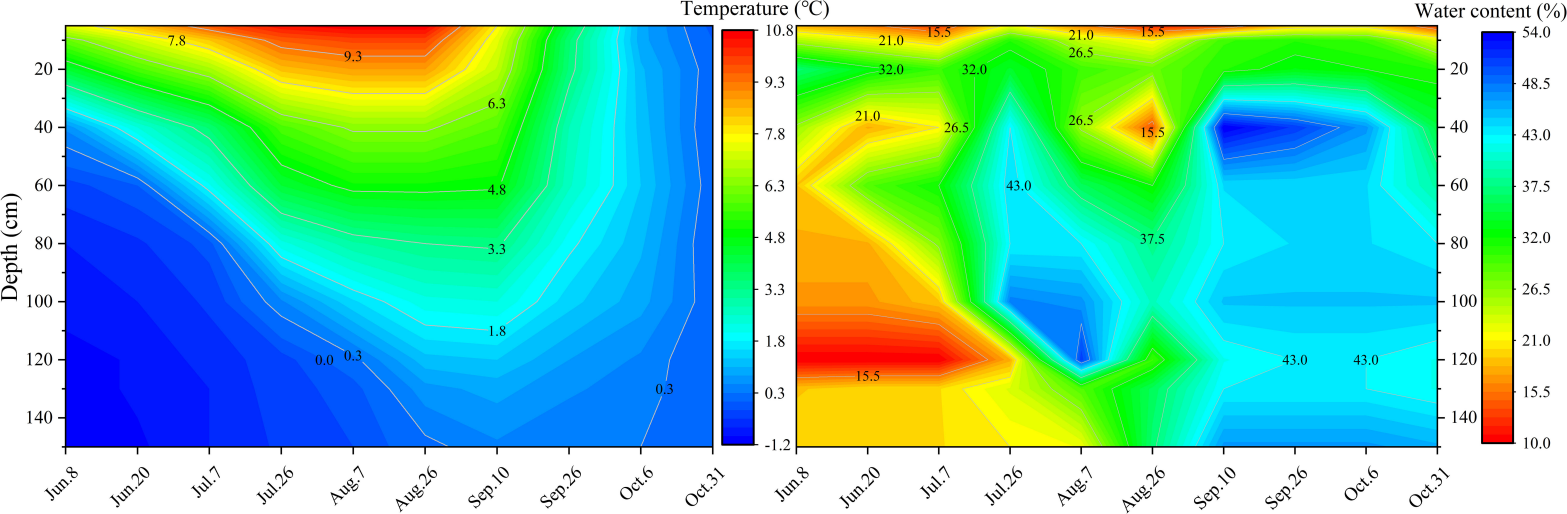
Changes in soil temperature and soil water content in BJ01.

From June to October, the soil temperature exhibited an initial increase followed by a subsequent decrease. Furthermore, the soil temperature gradually decreased with depth (**Figure 3**). Moderate changes were observed in the soil temperature isotherms from June 8 to August 26, while changes were more abrupt from September 10 to October 6. This indicates that the soil warming rate was much slower than the soil cooling rate.

From June 8 to August 7, the gradual increase in soil temperature and the downward progression of the melting front within the active layer of permafrost coincided with an incremental rise in soil water content at increasing depths. This resulted in substantial variations in the soil water content with a maximum fluctuation of 34.84% (**Figure 3**). Soil temperatures remained above 0°C, marking the cessation of thawing in the active layer of permafrost (Dong et al., 2021). The thawing of the active layer largely concluded between August 26 and October 6, the soil water content was approximately 32% in the surface layer and about 43% in the middle and deep layers (**Figure 3**). After mid-October, the active layer of permafrost gradually transitioned into a freezing state.

### 3.3 Precipitation, soil water and xylem water isotopes

**Figure 4:**
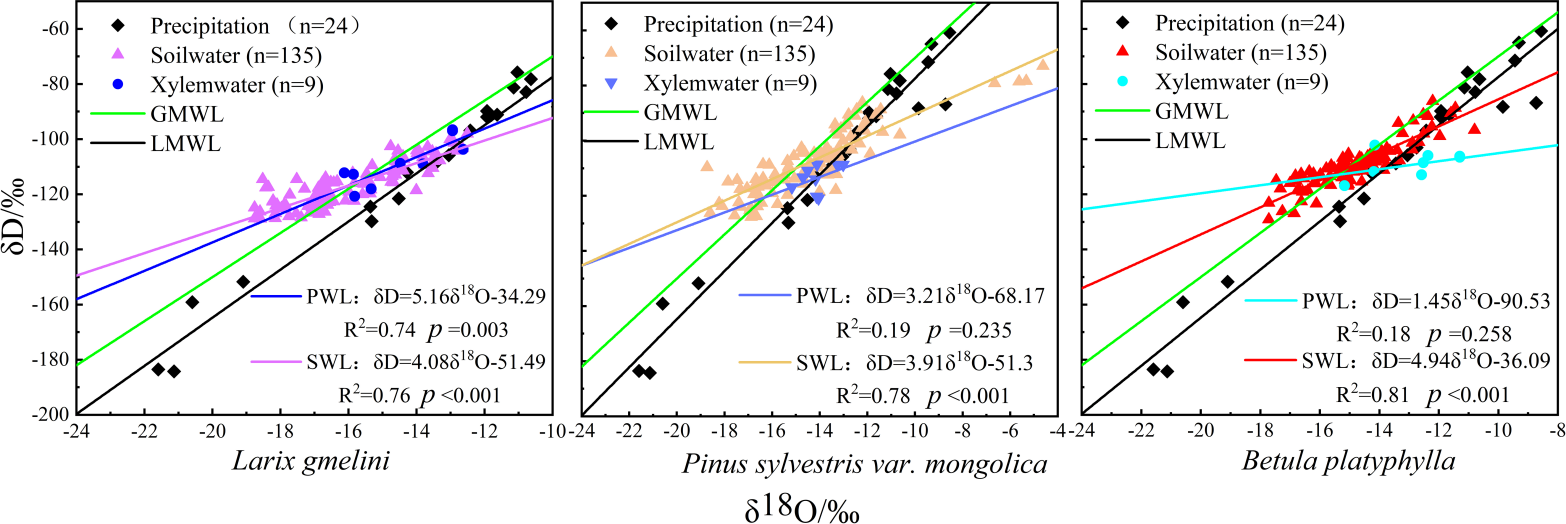
Distribution of xylem water, soil water and precipitation, δD and δ^18^O values for different tree species and corresponding linear regressions (GMWL – δD=8δ^18^O+10; LMWL – δD=8.73δ^18^O+9.85, R^2^=0.96, *p*<0.001; PWL – plant xylem water; SWL – soil water).

The δD values of precipitation samples varied from -183.55‰ to -60.77‰, averaging -106.01‰. Similarly, δ^18^O values spanned from -21.59‰ to -8.56‰, averaging -13.27‰. Overall, there was significant fluctuation and poor stability. The slope of the local atmospheric precipitation line (LMWL: δD=8.73δ^18^O+9.85, R^2^=0.96, *p*<0.001) exceed that of the global atmospheric precipitation line (GMWL: δD=8δ^18^O+10) (**Figure 4**), indicating greater isotopic fractionation and higher air humidity during the precipitation process (Xia et al., 2019).

**Figure 5:**
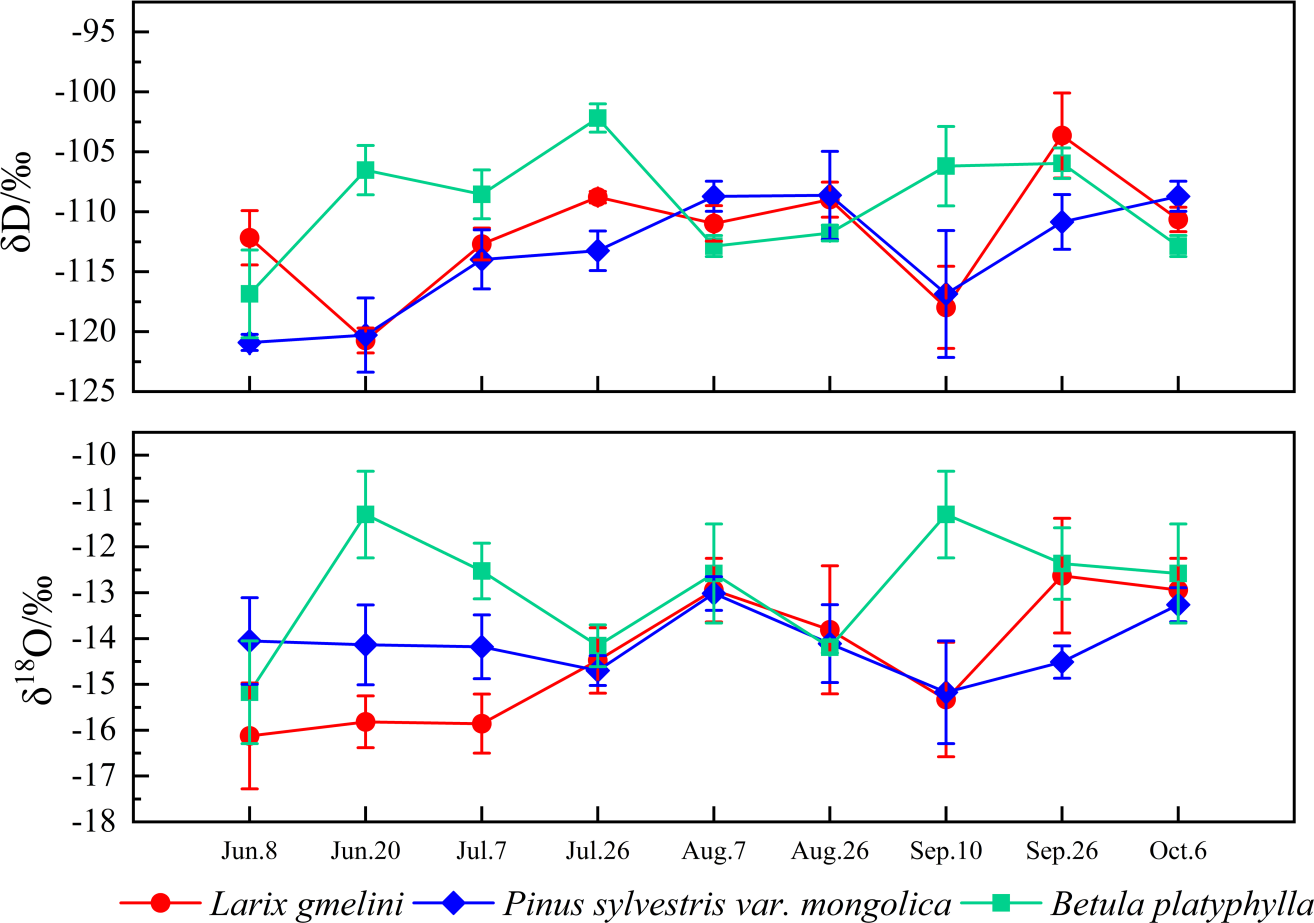
Variation of δD and δ^18^O values in xylem water of different trees.

Among the three vegetation species, the plant water line for *L. gmelinii* displayed the highest slope, while *B. platyphylla* exhibited the lowest (**Figure 4**). Compared to precipitation, the δD and δ^18^O values in the xylem water of the three vegetation species showed less variation and were closely aligned with the isotopic composition of their respective soil waters (**Figure 4**). The highest xylem water δD values were recorded in *B. platyphylla*, while the lowest were observed in *P. sylvestris*. Correspondingly, the highest δ^18^O values were measured in *B. platyphylla*, followed by *P. sylvestris* and *L. gmelinii* (**Figure 5**). There were no significant differences in xylem δD among the three tree species (p > 0.05). Additionally, the δ^18^O values of xylem water in the three vegetation species demonstrated similar patterns and maintained consistent trends on July 26, August 7, and August 26. However, during other periods, the magnitude of fluctuations varied.

**Figure 6:**
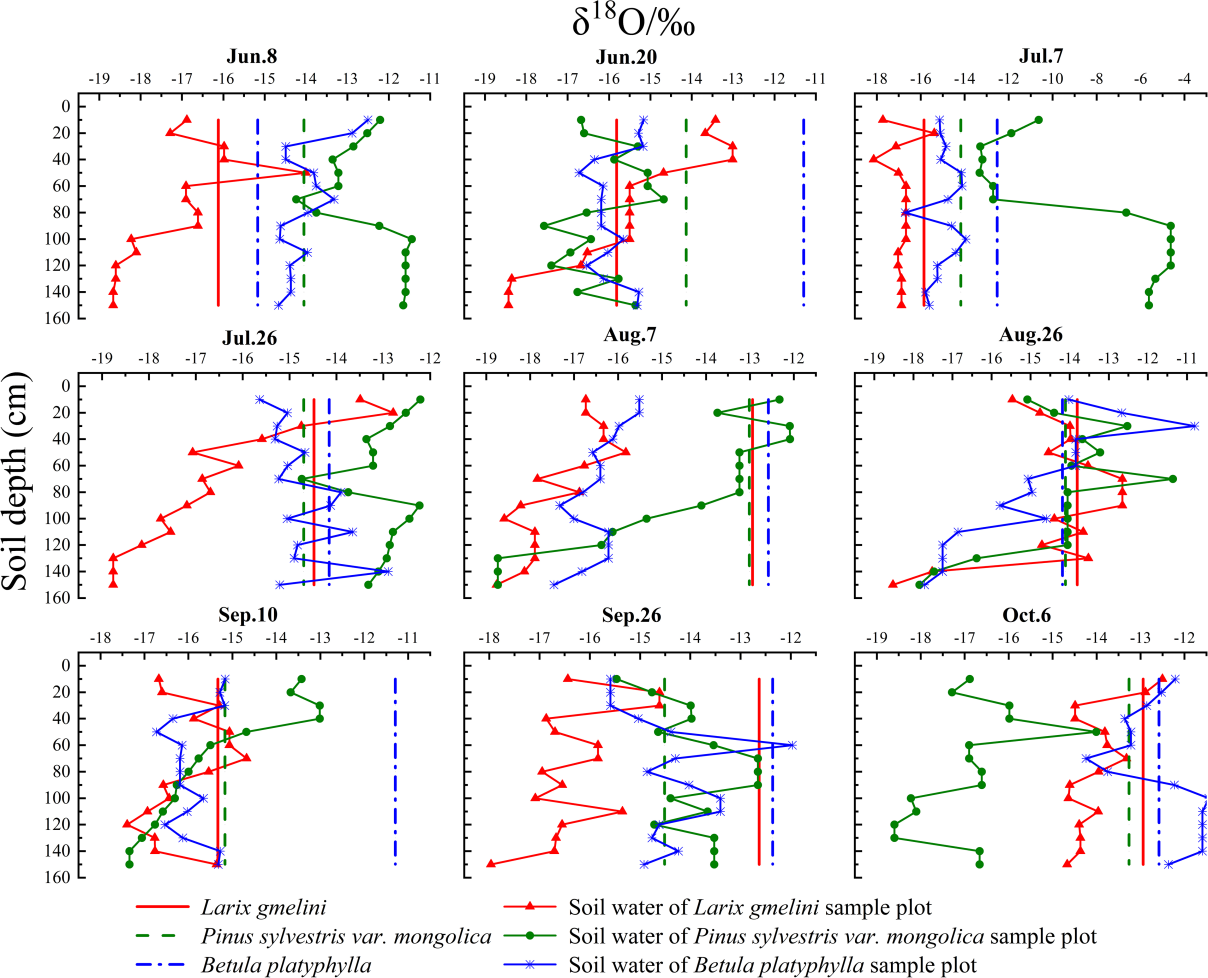
δ^18^O intersection of xylem of different tree species with their soil water.

The slope and intercept of the soil water lines of all three vegetation species were lower than those of the local atmospheric precipitation line, with *B. platyphylla* being the highest and *P. sylvestris* the lowest. *P. sylvestris* exhibited the highest average soil water δD and δ^18^O values, while the lowest average values were recorded in *L. gmelinii*. During the same period, the three vegetation species showed distinct differences in soil water δ^18^O values (**Figure 6**). The δ^18^O values within the soil profile showed enrichment in the shallow layer and decreased progressively with depth.

### 3.4 Water sources of three vegetation species

The vegetation water sources were analyzed based on direct comparisons. At points where the δ^18^O values of xylem water and soil water intersected, and their proximity was notable, these intersections were considered indicative of the primary water sources for vegetation (Laur and Hacke, 2014; Yang et al., 2015). Conversely, if no intersection occurred between the δ^18^O values of vegetation xylem water and soil water (e.g., absence of intersection for *L. gmelinii* on August 7), the primary water sources could not be conclusively identified (**Figure 6**). The *L. gmelinii* xylem water δ^18^O exhibited several intersections with soil shallow water (0-40 cm), while complex changes were observed for *P. sylvestris*. The xylem of *B. platyphylla* exhibited more intersections with the soil middle water layer (40-110 cm) compared to the other layers.

**Figure 7:**
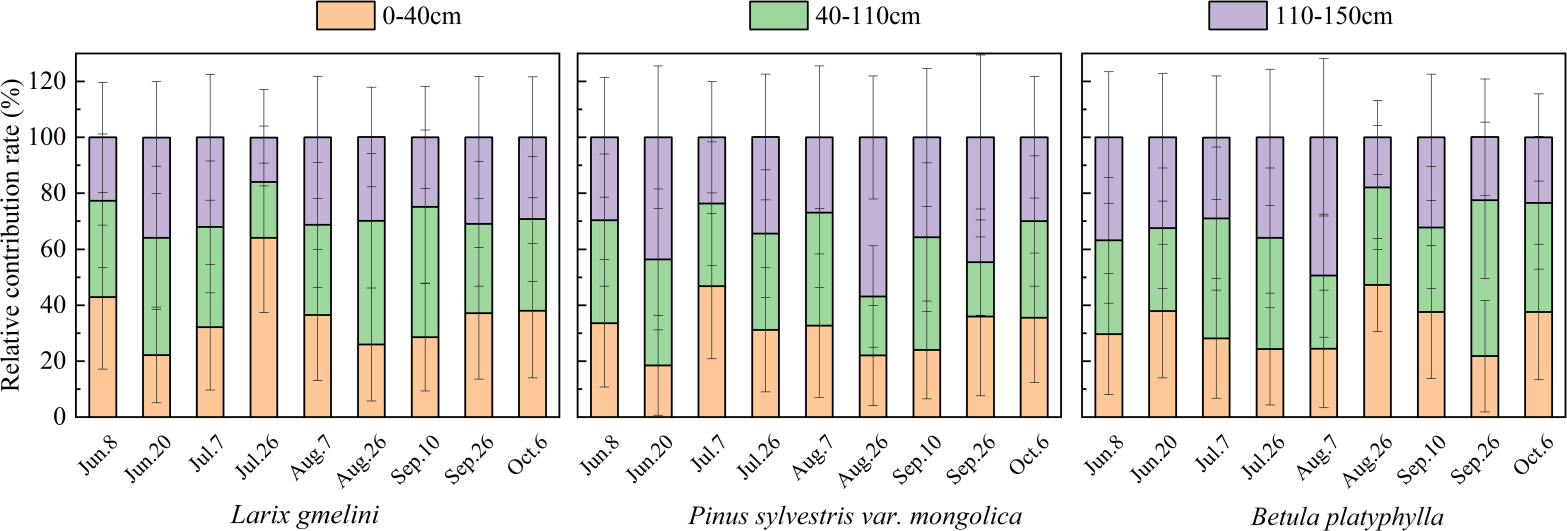
Contribution of soil water from different layers to the three tree species.

The results from the MixSIAR model and the direct comparisons essentially yielded the same conclusions for the main water absorption layer of vegetation (**Figure 7**). *L. gmelinii* primarily used shallow soil water (maximum contribution of 64.1%), *P. sylvestris* mainly used middle (maximum contribution of 40.4%) and deep (maximum contribution of 56.9%) soil water, and *B. platyphylla* generally relied on middle soil water (maximum contribution of 55.7%). Furthermore, *P. sylvestris* utilized deep soil water mainly on June 20, August 26 and September 26, with contributions of 43.6%, 56.9% and 44.6%, while the greatest utilization of deep soil water by *B. platyphylla* was observed on June 8 and August 7, with contributions of 36.8% and 49.4%. The results indicate that *P. sylvestris* has the greatest capacity to utilize deep soil water, followed by *B. platyphylla*, while *L. gmelinii* relies less on deep soil water.

## 4 Discussion

### 4.1 Soil water dynamics

From June to October, soil water content exhibited significant fluctuations, reaching up to 41%, in response to variations in soil temperature. From June 8 to August 7, soil water content progressively increased with depth (**Figure 3**). However, between June 8 and July 26, the soil water content at 100-120 cm was at its lowest. This phenomenon is mainly attributed to the soil’s limited water-holding capacity. Specifically, liquid water produced by the top-down thawing of the active layer, combined with precipitation, forms surface water that migrates downward to the soil layer near the upper limit of the permafrost due to gravitational forces (Romanovsky et al., 2010). Therefore, the soil water content at 100-120 cm was lower than that in both the overlying and underlying layers.

The relatively high soil water content at 0-20 cm on July 26 was primarily caused by the continuous rainfall prior to this date, which infiltrated into the shallow layer of the soil and migrated downward by gravity (Hinkel et al., 2001). Rainfall has been demonstrated to strongly affect soil (0-80 cm) water (Xu et al., 2011). The water content was high and relatively stable from September 10 to October 6. The active layer entered the freezing period from October 6 to October 31, resulting in a decrease in unfrozen soil water content. The active layer freezes in both directions and freezing occurs much faster than the thawing process (Dong et al., 2021).

### 4.2 Hydrogen and oxygen isotopic variability in diverse water bodies

The δD and δ^18^O values of the precipitation samples exhibited significant fluctuations and low stability. This conclusion is consistent with findings from numerous studies (Song et al., 2016; Wang et al., 2017). Song et al. (2016) observed variations in the water sources for *P. sylvestris*, noting significant fluctuations in the hydrogen and oxygen isotopes of precipitation (-3.6‰ to -17.0‰ for δ^18^O and -126.5‰ to -33.4‰ for δD) throughout the observation period. Wang et al. (2017) reported significant fluctuations in the hydrogen and oxygen isotopes of precipitation between sampling dates while studying seasonal changes in water absorption by three vegetation species. Variations in δD and δ^18^O in precipitation across different regions are likely associated with discrepancies in precipitation sources and meteorological conditions during different time periods (Hao and Li, 2021).

Compared to precipitation, variations in δD and δ^18^O values in vegetation xylem water exhibited markedly less variability. The isotopic composition of xylem water reflects the integration of multiple water sources, with seasonal fluctuations in these isotopic signatures indicating variations in water source utilization by vegetation across various growth periods (Laur and Hacke, 2014). In this study, the highest and lowest water δD values in the xylem were observed in *B. platyphylla* and *P. sylvestris*, respectively. *B. platyphylla* exhibited the highest δ^18^O values, followed by *P. sylvestris* and *L. gmelinii*. The disparities in hydrogen and oxygen isotope compositions across various vegetation species predominantly reflect the inherent traits of the vegetation (Hao and Li, 2021). Additionally, the δD and δ^18^O values in xylem water were very close to those in soil water (Wang et al., 2010), indicating that *L. gmelinii*, *P. sylvestris*, and *B. platyphylla* primarily obtained water from the soil during their growth.

During the same period, the three vegetation species exhibited distinct δ^18^O isotopic variations in soil water. Hao and Li (2021) suggested that the disparities in δD and δ^18^O values of soil water among various vegetation types might stem from differences in soil texture, structure, vegetation types, and the extent of community coverage. In the soil profiles of the three vegetation species, δ^18^O values were enriched in the upper layers and declined progressively with depth. This was mainly because the process of dynamic fractionation was more intense in the shallow soil layers, where lighter water molecules (^1^H_2_^16^O) evaporated first, enriching the δ^18^O values of shallow soil water. In contrast, the deep soil water was less influenced by environmental factors and exhibited weaker evaporation effects (Sprenger et al., 2016). The δD and δ^18^O values of soil water closely approximated the local atmospheric precipitation line, indicating substantial replenishment of soil water from rainfall (Wang et al., 2017; Huo et al., 2018). This study reveals that precipitation predominantly replenishes shallow soil water, with less impact on the middle and deep layers. The positioning of δD and δ^18^O values of soil water in the middle and deep layers above and to the left of the local precipitation line suggests that the water in these layers, which supports vegetation growth, primarily originates from the melting of the active permafrost layer.

### 4.3 Vegetation water sources

From June to October, *L. gmelinii* obtained roughly 71.9% of its water from the 0-110 cm soil layer, yet demonstrated a stronger dependence on the more superficial 0-40 cm layer. Similar results were found in other permafrost studies. For example, in the permafrost regions of Siberia, research indicated that 80% of the fine roots of *L. gmelinii* were located within the upper 30 cm of the soil profile. Notably, roots found between 30 and 70 cm depths were predominantly responsible for water uptake (Sugimoto et al., 2002). However, Churakova-Sidorova et al. (2020) showed that *L. gmelinii* does not obtain water beyond 50 cm of the soil. The observed variations in results may be attributed to the diverse root environments shaped by the thawing depth of the active permafrost layer and the soil structure across different regions, leading to unique vegetation water use strategies. Nonetheless, it is apparent that *L. gmelinii* has a greater reliance on water from shallow soil depths.

Compared to *L. gmelinii*, *P. sylvestris* is able to utilize deeper soil water (110-150 cm). This phenomenon can be ascribed to the more extensive root distribution exhibited by *P. sylvestris*. Plants with deep root systems are capable of accessing deep soil water and groundwater more effectively than those with shallow roots (Nie et al., 2011; Dai et al., 2015). Song et al. (2016) demonstrated that 98% of *P. sylvestris* roots were located at depths up to 1 meter. In spring and autumn, these trees predominantly utilized soil water from the upper 40 cm layer, while in summer, to accommodate increased transpiration, they accessed both deeper soil water (40-60 cm) and groundwater. In addition, water use patterns also vary with the tree age. For example, Huo et al. (2018) found that trees aged 15 and 22 years exhibited a higher utilization of deep soil water (80-200 cm) and groundwater compared to their younger counterparts aged 4 and 8 years. Given the greater robustness of the *P. sylvestris* samples compared to *L. gmelinii* and *B. platyphylla* (2.1), we speculate that the age of *P. sylvestris* is slightly higher than that of the latter species. This may also result in *P. sylvestris* being able to utilize deeper soil water.

*B. platyphylla* accessed soil water from the 0-110 cm layer at a rate of 68.9%, mirroring that of *L. gmelinii*, yet its exploitation of deeper soil layers exceeded that observed in *L. gmelinii*. Additionally, *B. platyphylla* showed a greater reliance on water from the intermediate soil layer (40-110 cm). Rao et al. (2020) demonstrated that in the cold-dry month of May, *B. platyphylla* accesses water from multiple soil depths up to 140 cm. However, during the warm-wet period of October, the species derives over 70% of its water from the 60-80 cm layer. Under conditions of adequate water supply, *B. platyphylla* predominantly extracts water from the middle soil layer. In comparison to *L. gmelinii*, it taps less into the deeper soil water resources.

Plants with dimorphic roots are capable of alternating their water source from shallow to deep soil layers (Nie et al., 2011; Yang et al., 2015). Additionally, the ability of the absorbent layer to facilitate transfer between these layers reflects the ecological plasticity of the vegetation (Valladares et al., 2007). Greater ecological plasticity correlates with enhanced environmental adaptability (Wang et al., 2017). From July to September, the water demand and transpiration of vegetation reach their maximum for the year due to higher temperatures. During these months, *L. gmelinii* primarily transitions between shallow and middle soil layers for water uptake, while *P. sylvestris* and *B. platyphylla* utilize water sources across shallow, middle, and deep layers. *P. sylvestris* demonstrates greater flexibility in shifting water absorption layers compared to *L. gmelinii* and *B. platyphylla*. Thus, we can conclude among the three tree species, *P. sylvestris* exhibits the highest ecological plasticity, followed by *B. platyphylla*, with *L. gmelinii* displaying the lowest. More specifically, *P. sylvestris* exhibits superior environmental adaptability compared to the other two species. Moreover, precipitation substantially impacts the water absorption capabilities of vegetation (Wu et al., 2015). Continuous rainfall before the sampling date of July 26 caused the water uptake for the three tree species to be largely confined to the shallow and middle layers of the soil. Notably, the water uptake of *L. gmelinii* was the most pronounced, consuming up to 64.1% of the water from the shallow layer.

### 4.4 Implications of water sources for different species

In recent years, the susceptibility of tree growth to temperature has decreased in relatively humid boreal forests (Briffa et al., 1998). This may be attributed to the greater water demand of plants due to global warming (Barber et al., 2000; Sugimoto et al., 2002; Grube et al., 2017). In permafrost regions, permafrost degradation accelerates water infiltration and evaporation, disrupting the hydrological cycle between vegetation and permafrost and increasing water stress on vegetation (Chen et al., 2022). As a dominant tree in the permafrost regions of the Greater Khingan Mountains, *L. gmelinii* has shallow roots and a high demand for shallow soil water (Sugimoto et al., 2002). Currently, the permafrost thawing period provides sufficient water during its growing season. However, over time, ongoing permafrost degradation leads to deeper thawing of the active layer, causing soil water to migrate downward under gravity, while water in the surface soil is subject to evaporation. When the soil can no longer provide sufficient water for *L. gmelinii*, its roots may extend deeper, or it may gradually be replaced by other species. Consequently, the growth of *L. gmelinii* is greatly affected, and this view is supported by numerous studies. Since the 1980s, the radial growth of *L. gmelinii* has significantly reduced (Chen et al., 2022). Moreover, the degradation of the permafrost can cause a large loss of nutrients in the soil, substantially affecting the growth of *L. gmelinii* (Bai et al., 2016). The degradation of the permafrost may thus lead to the decline of *L. gmelinii* (Chen et al., 2022). *P. sylvestris* exhibits low water demand and transpiration rates, enabling it to adapt to environmental changes by modulating its water absorption across different soil layers (Sugimoto, 2001). Furthermore, *P. sylvestris* is less affected by permafrost degradation and can survive in dry climates (Sugimoto et al., 2002). Based on our results, we predict that permafrost degradation can release water and nutrients, which may favor vegetation growth. Over time, the three tree species will be affected to different degrees, with *L. gmelinii* receiving the greatest impact and *P. sylvestris* the least. However, this study has certain limitations. Isotopic fractionation may occur during soil water uptake by vegetation, potentially biasing the results of the direct comparison method. While the MixSIAR model can handle more complex data, it may introduce uncertainty in the determination of end-members.

## 5 Conclusion

In this study, the isotopic signatures of hydrogen and oxygen were used to determine the water sources of three vegetation species, namely, *L. gmelinii*, *P. sylvestris* and *B. platyphylla*, from June to October 2019, in the Greater Khingan Mountains. The results revealed that *L. gmelinii* primarily utilized shallow soil water, *B. platyphylla* generally accessed middle soil water, and *P. sylvestris* mainly relied on both middle and deep soil water. *P. sylvestris* was identified as being more capable of adapting to environmental changes. Permafrost degradation was suggested to have the greatest impact on *L. gmelinii*, while *P. sylvestris* was observed to be the least affected. This study provides valuable insights into the impact of permafrost degradation on three vegetation species.

## Data Availability

The original contributions presented in the study are included in the article/supplementary material. Further inquiries can be directed to the corresponding author.
